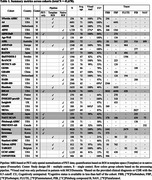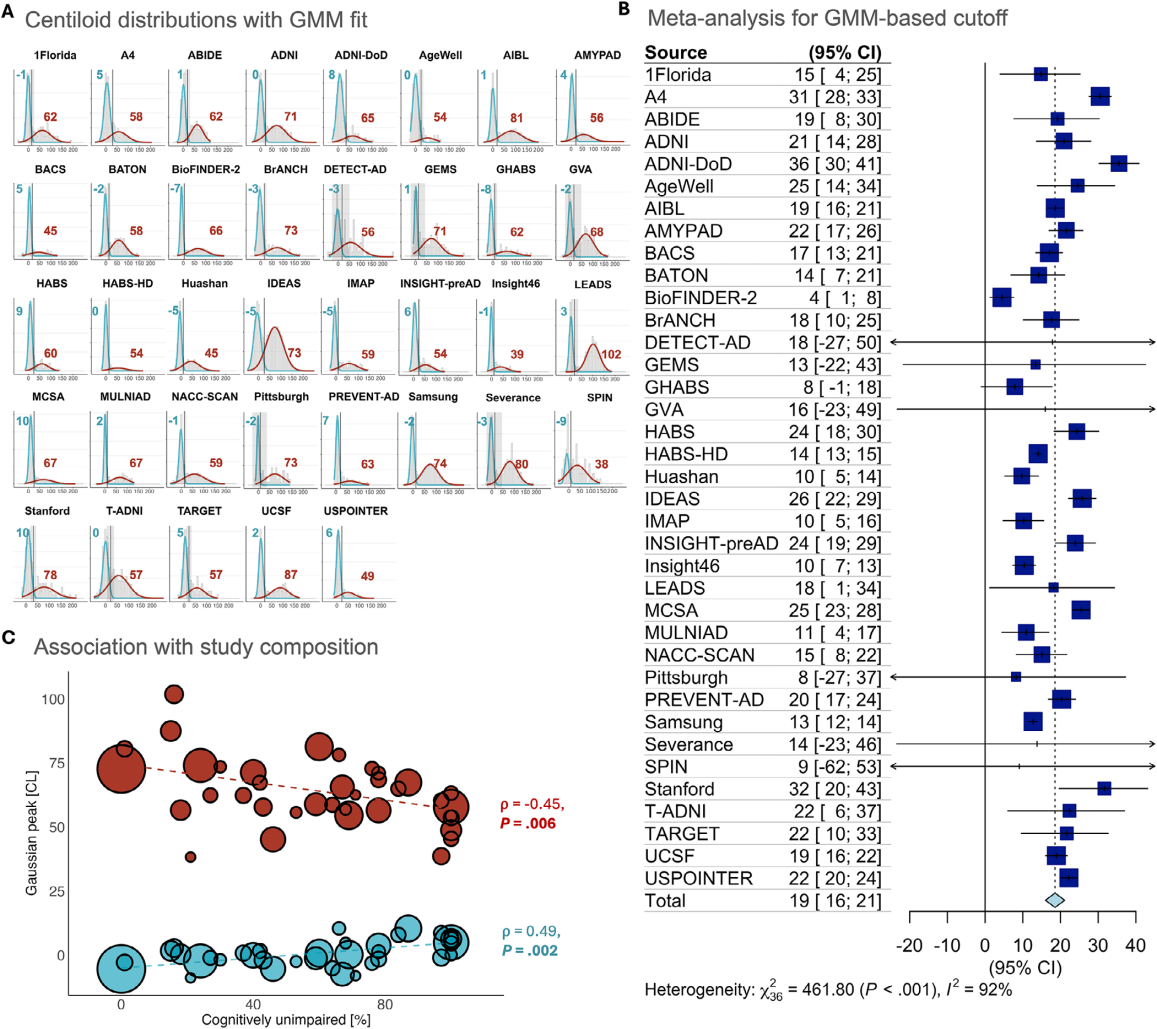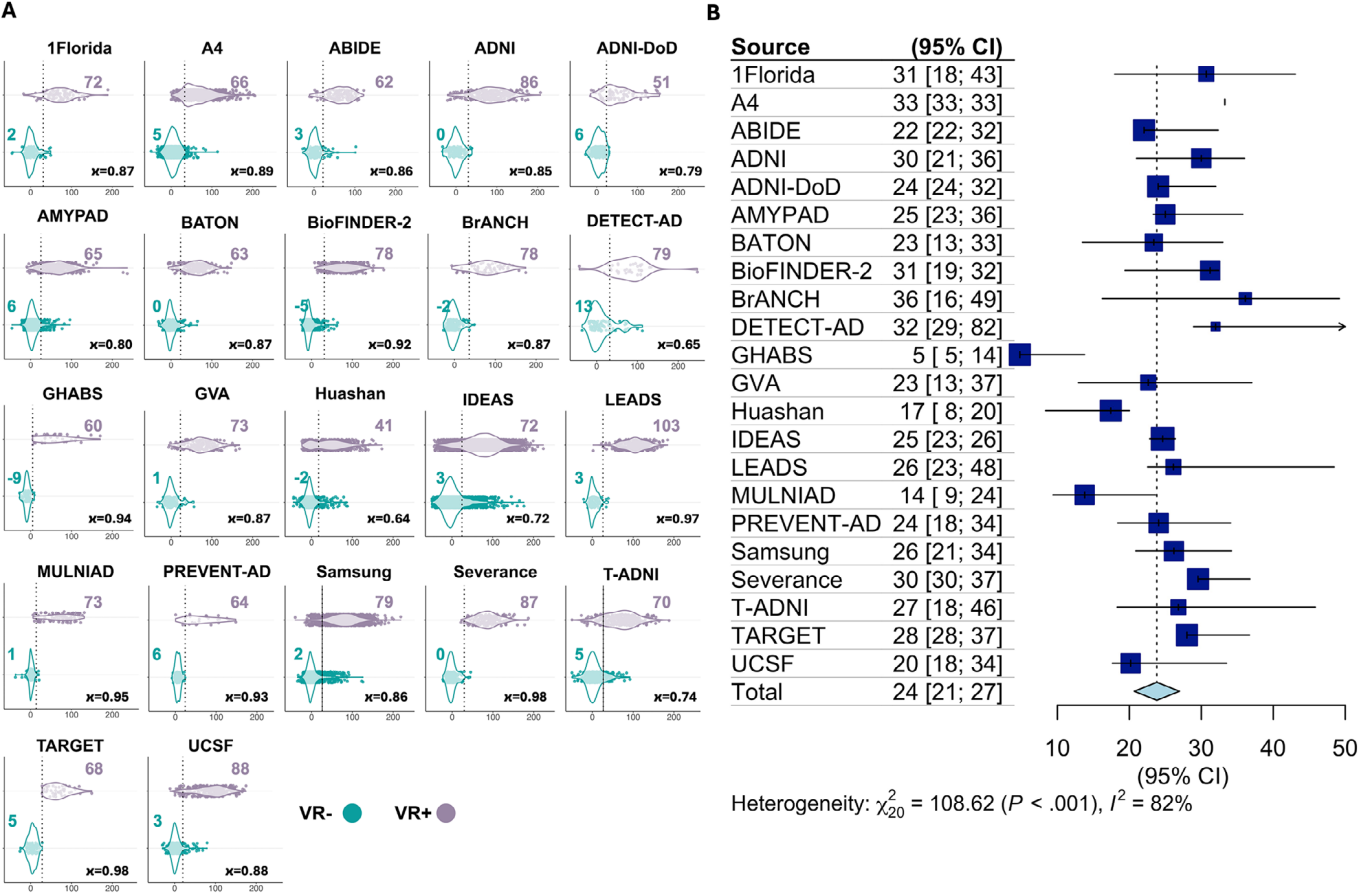# Every Centiloid, from Everywhere, All at Once

**DOI:** 10.1002/alz70856_107124

**Published:** 2026-01-08

**Authors:** Ganna Blazhenets, Heather M Snyder, Liana G. Apostolova, Breton M. Asken, Alexandre Bejanin, Stéphanie Bombois, Pierrick Bourgeat, Meredith N. Braskie, Maria C. Carrillo, Kaitlin B Casaletto, David M Cash, Chiung‐Chih Chang, Hsin‐I Chang, Yishu Chao, Gael Chételat, William Coath, Lyduine E. Collij, Lise Colmant, Brad C. Dickerson, Bruno Dubois, Alfonso Fajardo Valdez, Gill Farrar, Juan Fortea, Giovanni B Frisoni, Raquel C Gardner, Valentina Garibotto, Yuna Gu, Tengfei Guo, Bernard J Hanseeuw, Oskar Hansson, Theresa M. Harrison, Qi Huang, Shu‐Hua Huang, Kazunari Ishii, Kenji Ishii, William J. Jagust, Takashi Kato, Robert A. Koeppe, Joel H Kramer, Susan M. Landau, Brigitte Landeau, Sangwon Lee, Brian J Lopresti, Val J Lowe, Xiaoxie Mao, Andrew March, Colin L. Masters, Florence Mézenge, Elizabeth C. Mormino, Maria Franquesa‐Mullerat, Akinori Nakamura, Sid E. O'Bryant, Ioannis Pappas, Débora E. Peretti, Lisa Quenon, Christopher C. Rowe, Gemma Salvadó, Jonathan M Schott, Daniel Schwartz, Christopher G Schwarz, Sang Won Seo, Mahnaz Shekari, Lisa C Silbert, Ruben Smith, Andrzej Sokolowski, David N. Soleimani‐Meigooni, Reisa A. Sperling, Pan Sun, Arthur W. Toga, David E. Vaillancourt, Elsmarieke van de Giessen, Wiesje M. van der Flier, Prashanthi Vemuri, Nicolas Villain, Victor L. Villemagne, Sylvia Villeneuve, Wei‐En Wang, Michael S. W. Weiner, Cally Xiao, Fang Xie, Yeojun Yoon, Christina B. Young, Mijin Yun, Gil D. Rabinovici, Renaud La Joie, AMYPAD and LEADS Consortia

**Affiliations:** ^1^ Memory and Aging Center, Weill Institute for Neurosciences, University of California San Francisco, San Francisco, CA, USA; ^2^ Alzheimer's Association, Chicago, IL, USA; ^3^ Department of Neurology, Indiana University School of Medicine, Indianapolis, IN, USA; ^4^ 1Florida Alzheimer's Disease Research Center, Department of Clinical and Health Psychology, University of Florida, Gainesville, FL, USA; ^5^ Sant Pau Memory Unit, Department of Neurology, Hospital de la Santa Creu i Sant Pau, Institut d'Investigació Biomèdica Sant Pau (IIB SANT PAU), Facultad de Medicina ‐ Universitat Autònoma de Barcelona, Barcelona, Spain; ^6^ Center of Biomedical Investigation Network for Neurodegenerative Diseases (CIBERNED), Madrid, Spain; ^7^ Institute of Memory and Alzheimer's Disease (IM2A), Department of Neurology, Pitié‐Salpêtrière Hospital, Paris, France; ^8^ Florey Institute of Neuroscience and Mental Health, University of Melbourne, Melbourne, VIC, Australia; ^9^ Mark and Mary Stevens Neuroimaging and Informatics Institute, Keck School of Medicine, University of Southern California, Los Angeles, CA, USA; ^10^ Memory and Aging Center, UCSF Weill Institute for Neurosciences, University of California, San Francisco, San Francisco, CA, USA; ^11^ Dementia Research Centre, UCL Queen Square Institute of Neurology, University College London, London, United Kingdom; ^12^ Department of Neurology, Cognition and Aging Center, Institute for Translational Research in Biomedicine, Kaohsiung Chang Gung Memorial Hospital, Chang Gung University College of Medicine, Kaohsiung, Taiwan; ^13^ Department of Medicine, National Sun Yat‐Sen University, Kaohsiung, Taiwan; ^14^ University of California, Berkeley, Berkeley, CA, USA; ^15^ Normandie Université, Université de Caen, Institut National de la Santé et de la Recherche Médicale, Caen, France; ^16^ Department of Radiology and Nuclear Medicine, Vrije Universiteit Amsterdam, Amsterdam University Medical Center, location VUmc, Amsterdam, Netherlands; ^17^ Clinical Memory Research Unit, Department of Clinical Sciences Malmö, Faculty of Medicine, Lund University, Lund, Sweden; ^18^ Department of Neurology Cliniques universitaires Saint‐Luc, Brussel, Belgium; ^19^ Department of Neurology, Massachusetts General Hospital, Harvard Medical School, Boston, MA, USA; ^20^ Department of Psychiatry, McGill University, Montréal, QC, Canada; ^21^ GE HealthCare, Chalfont St Giles, Buckinghamshire, United Kingdom; ^22^ Laboratory of Neuroimaging of Aging (LANVIE), University of Geneva, Geneva, Switzerland; ^23^ Geneva Memory Center, Department of Rehabilitation and Geriatrics, Geneva University Hospitals, Geneva, Switzerland; ^24^ Joseph Sagol Neuroscience Center, Sheba Medical Center, Ramat Gan, Israel; ^25^ Division of Nuclear Medicine and Molecular Imaging, Geneva University Hospitals, Geneva, Switzerland; ^26^ Centre for Biomedical Imaging, University of Geneva, Geneva, Switzerland; ^27^ Laboratory of Neuroimaging and Innovative Molecular Tracers (NIMTlab), Geneva University Neurocenter and Faculty of Medicine, University of Geneva, Geneva, Switzerland; ^28^ Samsung Medical Center, Sungkyunkwan University School of Medicine, Gangnam‐gu, Seoul, Korea, Republic of (South); ^29^ Institute of Biomedical Engineering, Peking University Shenzhen Graduate School, Peking, China; ^30^ Clinical Memory Research Unit, Lund University, Malmö, Skåne, Sweden; ^31^ Neuroscience Department, University of California, Berkeley, Berkeley, CA, USA; ^32^ Department of Nuclear Medicine & PET Center, Huashan Hospital, Fudan University, Shanghai, Shanghai, China; ^33^ Department of Nuclear medicine, Kaohsiung Chang Gung Memorial Hospital, Chang Gung University College of Medicine, Kaohsiung, Taiwan; ^34^ Faculty of Medicine, Kindai University, Osakasayama, Osaka, Japan; ^35^ Tokyo Metropolitan Institute for Geriatrics and Gerontology, Tokyo, Tokyo, Japan; ^36^ National Center for Geriatrics and Gerontology, Obu, Aichi, Japan; ^37^ University of Michigan, Ann Arbor, MI, USA; ^38^ Department of Nuclear Medicine, Yonsei University College of Medicine, Seoul, Korea, Republic of (South); ^39^ University of Pittsburgh, School of Medicine, Pittsburgh, PA, USA; ^40^ Department of Radiology, Mayo Clinic, Rochester, MN, USA; ^41^ American College of Radiology, Reston, VA, USA; ^42^ Department of Neurology and Neurological Sciences, Stanford University School of Medicine, Stanford, CA, USA; ^43^ Institute for Translational Research, University of North Texas Health Science Center, Fort Worth, TX, USA; ^44^ Laboratory of Neuro Imaging, Stevens Neuroimaging and Informatics Institute, Keck School of Medicine, University of Southern California, Los Angeles, CA, USA; ^45^ Barcelonaβeta Brain Research Center (BBRC), Pasqual Maragall Foundation, Barcelona, Spain; ^46^ NIA‐Layton Aging & Alzheimer's Disease Center, Portland, OR, USA; ^47^ Samsung Medical Center, Gangnam‐gu, Seoul, Korea, Republic of (South); ^48^ Imaging Genetics Center, Mark and Mary Stevens Neuroimaging and Informatics Institute, Keck School of Medicine, University of Southern California, Marina del Rey, CA, USA; ^49^ 1Florida Alzheimer's Disease Research Center, Department of Applied Physiology and Kinesiology, University of Florida, Gainesville, FL, USA; ^50^ University of Pittsburgh School of Medicine, Pittsburgh, PA, USA

## Abstract

**Background:**

Ten years after the original publication, the Centiloid framework is now broadly used to harmonize amyloid‐PET quantification, facilitate data sharing and comparison across cohorts, and even assist visual interpretation in clinical settings. We evaluated the global implementation of Centiloids by comparing their distribution and corresponding positivity thresholds across cohorts.

**Methods:**

We gathered data from publicly available cohorts and reached out to investigators across the world to collect cross‐sectional Centiloids, demographic and clinical information, and visual read data. Gaussian mixture models (GMM, k=2) were fitted to Centiloid values for each cohort and cutoffs were calculated as mean + 2SD of the lower Gaussian. When visual reads were available, we determined Centiloid cutoffs that maximized correspondence with visual reads (Cohen's kappa). Data was combined across cohorts using random effects meta‐analyses.

**Results:**

As of January 2025, we included 37 cohorts (*n* = 41,678 participants) with heterogeneous pipelines, radiotracers, and clinical and demographic characteristics (Table‐1). The low Gaussian peaks ranged from ‐9 to 10CL; the meta‐analysis identified a common peak at 1CL. The second peaks were more heterogeneous (range=38‐102CL; meta‐analysis outcome=64CL). Across cohorts, the proportion of cognitively unimpaired versus impaired participants impacted the position of both peaks, with better separation in cohorts enriched in impaired individuals (Figure 1C). The meta‐analysis indicated a GMM‐based cutoff of 19CL (95%CI: 16‐21CL, Figure 1B); subgroup analyses showed no evidence of significant effect between single versus multicenter settings (17 versus 20CL, *p* = 0.30), MRI‐based or PET‐only processing (18 versus 19CL, *p* = 0.88), and no evidence of difference across radiotracers (Flutemetamol: 16CL; PIB: 17CL, Flutafuranol: 18CL, Florbetaben: 19CL, Florbetapir: 20CL, *p* = 0.78). In a subset of 29,496 participants with visual reads available, binary visual reads corresponded well to Centiloids (common kappa=0.86, Figure 2A). The visual read‐based cutoff of 24CL (95%CI: 21‐27CL, Figure 2B) maximized correspondence between visual read and quantification and was slightly higher than the GMM‐based cutoff. All meta‐analysis models showed high non‐random heterogeneity (I^2^>80%) across studies, suggesting non‐random differences in peaks and cutoffs.

**Conclusions:**

Meta‐analysis‐based cutoffs align well with thresholds from the existing literature. High heterogeneity among studies underscores the need to investigate contributing factors, raising concerns about applying common cutoffs.